# A Multi-Feature Fusion Framework for Automatic Skin Cancer Diagnostics

**DOI:** 10.3390/diagnostics13081474

**Published:** 2023-04-19

**Authors:** Samy Bakheet, Shtwai Alsubai, Aml El-Nagar, Abdullah Alqahtani

**Affiliations:** 1 Faculty of Computers and Artificial Intelligence, Sohag University, Sohag 82524, Egypt; samy.bakheet@fci.sohag.edu.eg (S.B.);; 2 Institute for Information Technology and Communications (IIKT), Otto-von-Guericke-University Magdeburg, D-39106 Magdeburg, Germany; 3 College of Computer Engineering and Sciences, Prince Sattam bin Abdulaziz University, Al Kharj 11942, Saudi Arabia

**Keywords:** malignant melanoma, skin cancer, computer-aided diagnosis, histogram of oriented gradient, local binary patterns, support vector machine, k-nearest neighbors, gentle adaboost, cross-validation

## Abstract

Malignant melanoma is the most invasive skin cancer and is currently regarded as one of the deadliest disorders; however, it can be cured more successfully if detected and treated early. Recently, CAD (computer-aided diagnosis) systems have emerged as a powerful alternative tool for the automatic detection and categorization of skin lesions, such as malignant melanoma or benign nevus, in given dermoscopy images. In this paper, we propose an integrated CAD framework for rapid and accurate melanoma detection in dermoscopy images. Initially, an input dermoscopy image is pre-processed by using a median filter and bottom-hat filtering for noise reduction, artifact removal, and, thus, enhancing the image quality. After this, each skin lesion is described by an effective skin lesion descriptor with high discrimination and descriptiveness capabilities, which is constructed by calculating the HOG (Histogram of Oriented Gradient) and LBP (Local Binary Patterns) and their extensions. After feature selection, the lesion descriptors are fed into three supervised machine learning classification models, namely SVM (Support Vector Machine), kNN (k-Nearest Neighbors), and GAB (Gentle AdaBoost), to diagnostically classify melanocytic skin lesions into one of two diagnostic categories, melanoma or nevus. Experimental results achieved using 10-fold cross-validation on the publicly available MED-NODEE dermoscopy image dataset demonstrate that the proposed CAD framework performs either competitively or superiorly to several state-of-the-art methods with stronger training settings in relation to various diagnostic metrics, such as accuracy (94%), specificity (92%), and sensitivity (100%).

## 1. Introduction

Statistically speaking, cancer still remains among the leading causes of death worldwide, accounting for approximately 10 million deaths in 2020, or almost 1 in 6 deaths. According to the American Cancer Society, 120,774 deaths from skin cancer, 1,796,144 from lung cancer, 684,996 from breast cancer, 375,304 from prostate cancer, and 251,329 deaths from brain cancer are estimated in 2020 [1]. Skin cancer is the most common cancer in the United States and worldwide, which is divided mainly into two major categories, namely melanoma and non-melanoma skin cancer [2]. In spite of the fact that melanoma still represents less than 5% of all cutaneous malignancies, it accounts for a large majority of the deaths from skin cancer. According to the American Cancer Society, approximately 99,780 new cases of melanoma will be diagnosed and approximately 7650 individuals will die from the disease in 2022. Malignant melanoma is the most aggressive form of skin cancer and its prevalence has grown most rapidly worldwide particularly over the past few decades.

White people usually develop malignant melanoma due to excessive exposure to sunlight. The prognosis for melanoma patients is inversely proportional to the thickness of the tumor. Namely, as the thickness of the tumor increases, the survival rate decreases. Melanoma can usually be detected by simple observation, because it is confined to the skin. However, it is more likely to spread to the lymph nodes and, thus, increase the tumor malignancy. The large-scale systematic research in this area, and the advances in computer diagnostics, began in the 1980s, although early attempts have been emerging since the 1960s. Due to the rapid growth in computer vision technologies for analyzing medical imaging data, a broad range of research has been (and is still being) performed to contribute to improved diagnosis and prognosis of diseases [3,4]. The initial diagnosis of melanoma depends primarily on the patient’s alertness and practical evaluation by a medical practitioner. There are enormous diagnostic differences in addition to the deficient knowledge of test methods [5].

An exceptionally valuable technique for the diagnosis of melanotic lesions is Epiluminescence Microscopy (ELM), also known as skin-surface microscopy or ‘Dermatoscopy’, that is well-established as a reliable diagnostic tool to assist dermatologists and plastic surgeons to improve the accuracy of diagnosing melanoma and pigmented lesions [6]. Dermoscopy requirements involve high magnification and visualization of subsurface structures and patterns [7]. Recently, automated CAD systems that have been developed in the form of innovative web or mobile phone applications for early skin cancer diagnosis represent a potential emerging trend in the CAD software industry. The images input to these applications are typically acquired by digital cameras or video camera and frame grabber combinations.

In this work, we present a new framework for a CAD system for detecting malignant melanoma from dermoscopy images, which involves four main computational steps: pre-processing, segmentation, feature extraction, and classification of skin cancer into the categories malignant or benign. Basically, the initial step involves image preprocessing, such as image resizing and artifact and noise removal, to enhance the quality of the image. Moreover, an adaptive median filter is employed to remove artifacts and specific types of dermoscopic noise, such as uneven illumination, black frame, thin hair, bubbles, skin texture, etc. After enhancing the contrast and details of an input dermoscopic image, a uniform-distribution-based segmentation technique based on bottom-hat filtering is applied. In the second step, feature extraction, we propose to construct four feature descriptors, namely LBP and HOG and their modifications, for higher feature descriptiveness. After feature selection, the final feature descriptors are fed into three machine-learning models, namely SVM, kNN, and GAB, to diagnostically classify uncertain skin lesions as either melanoma or nevus.

The remainder of this paper is structured as follows: Section 2 reviews related prior work. The proposed CAD system framework is presented in Section 3. In Section 4, the experimental results are presented and discussed. Finally, in Section 5, the conclusions are given and possible directions of future work are pointed out.

## 2. Related Work

In recent years, automated CAD systems that aim to consolidate the automation of the disease diagnostic process have received a great deal of attention from researchers interested in the areas of pattern recognition and medical image processing, due to the development of various artificial intelligence and machine learning techniques. More specifically, with the development of many machine learning techniques, numerous image-based CAD systems have been developed for the screening and early detection of malignant melanoma in recent years [8]. Following recent technological and application trends, one can identify several emerging research and development areas in which a large number of researchers have greatly contributed to the current diversity in CAD approaches and techniques that help dermatologists in automatic melanoma detection from dermoscopic/non-dermoscopic images of pigmented skin lesions [9]. Accurate feature extraction of segmented skin lesions is a pivotal process in implementing well-established algorithms for CAD systems for extracting effective features that can reliably discriminate between different medical tissues.

Broadly speaking, automated CAD systems are mainly developed based on various feature descriptors, including shape, contrast, texture, and color [10]. The color features are widely used as a significant descriptor for melanoma detection [11], whereas the texture features derived using wavelet-decomposition are often combined with the border features derived from constructing a boundary series model of the skin lesion [12]. Furthermore, for representing and classifying dermoscopic images of skin lesions accurately and efficiently, several multi-modal CAD approaches employ multi-modal feature fusion strategies using Bag of Features (BoF) to enrich the feature representation and improve the robustness of features [13]. On reviewing the literature, a great deal of work has been conducted in relation to digital skin diagnostics to categorize melanoma images based on dermoscopic image characteristics. For instance, in [14], the authors made a comparison of six segmentation methods for skin lesions using dermoscopic images. Moreover, in [15], an effective framework of a CAD system for melanoma skin cancer is proposed, by the application of an SVM model on a set of optimized HOG-based features.

In another related work [16], the authors introduce an automated ABCD-rule based approach for detecting melanoma skin cancer, where the ABCD rule has been first developed in clinical diagnosis to discriminate benign from malignant melanoma lesions. The detection process was performed on images taken with a mobile device camera and the testing process was performed on the mobile.

In [17], an enhanced strategy for skin lesion detection is suggested, based on uniform segmentation and feature selection approach, which integrates preprocessing, lesion segmentation, features extraction, features selection, and classification. A serial-based method is applied subsequently to extract and fuse the traits, such as color, texture, and HOG shape. Then, the fused features are selected by implementing a Boltzman entropy technique and classified by SVM. The method was evaluated on the publicly available PH2 dataset, achieving promising results of sensitivity 97.7%, specificity 96.7%, accuracy 97.5%, and F-score 97.5%, which are significantly better than those of existing methods in the literature. Additionally, in [5], Bakheet and El-Nagar present a deep neural network (DNN)-based framework for real-time fine-grained classification and grading in dermoscopic skin cancer images, in which a compact set of visual features is extracted based on both color and typical geometric properties of skin lesions. The selected lesion features are then fed into a rapid DNN classifier for classifying lesions as melanoma or benign nevus. On the publicly available PH2 dermoscopy imaging dataset, the method was tested, achieving 97.5%, 96.67%, and 100.0% for diagnostic accuracy, sensitivity, and specificity, respectively, which compared favorably with those obtained from several state-of-the-art approaches. In a more recent study [18], the authors present an innovative skin lesion analysis technique for melanoma detection using a fuzzy deep learning GrabCut-Stacked Convolutional Neural Network (GC-SCNN) model, where deep learning algorithms [19] are incorporated and the fuzzy GC-SCNN model is applied for image training to automatically detect melanoma in dermoscopic images. Skin lesion features are extracted and classified from different publicly available dermoscopic image datasets. A hybrid model of fuzzy GC-SCNN with SVM achieved optimal results (99.75% for classification accuracy and 100% for both sensitivity and specificity). In addition, in [3], an effective CAD method for real-time melanoma detection is proposed, in which a low-dimensional yet discriminative descriptor for skin lesions is derived from local patterns of Gabor-based entropic features. The detection method was validated on the public PH2 benchmark dataset using 5-cross-validation, achieving 97.5%, 100%, and 96.87% in terms of accuracy, sensitivity, and specificity, respectively.

## 3. Proposed Methodology

**Figure 1:**
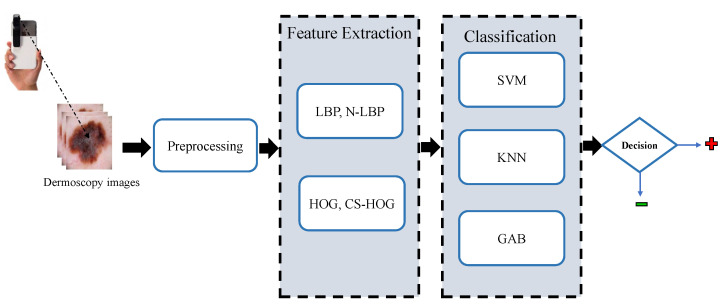
Block diagram of the proposed CAD system for skin lesions.

In this section, the proposed methodology for the automatic detection of melanoma skin cancer from dermoscopic images, which performs the automated diagnosis task in a series of steps, namely, preprocessing, lesion segmentation, feature extraction, and classification, is explained in detail. These steps are depicted in the block diagram shown in Figure 1 and as further elaborated in the following subsections.

### 3.1. Image Preprocessing

Image preprocessing plays a crucial role in constructing a stand-alone CAD system, due to its potential to improve the quality of skin lesion images by alleviating inherent cutaneous artifacts (e.g., hair and blood vessels) and undesirable artifacts (e.g., noise, air bubbles, color charts, marker ink, ruler marks, and vignette) that can basically make segmentation of skin lesions even more challenging. In the preprocessing stage of the proposed CAD system for skin lesions, an efficient scheme is applied for removing image abnormalities (e.g., hairs) from dermoscopic images, by employing a median filter followed by bottom-hat filtering to segment skin lesions from low-contrast noisy images. Proper selection of the structuring element is critical in morphological operations and it has to be carried out based on the image shape. Due to predominately round-shaped nature of the majority of skin lesion regions in non-dermoscopic images, morphological operations with a circular kernel as a structuring element (SE) are typically applied.

Prior to the skin lesion segmentation, the median filtering is specially performed to reduce impulsive salt–pepper noise in dermoscopy images, while contrast information is retained. The non-linear statistical approach of median filtering is used, which is widely used in medical image analysis and has been acknowledged as the best and most successful method of reducing noise from images [20]. In this process, a ‘window’ that represents the pattern of neighbors, moves pixel by pixel across the entire image, replacing the center value in the window with the median value of nearby pixels. The median is derived simply by sorting all of the pixel values in the window into numerical order, then the value of the pixel under consideration is replaced with the median value.

**Figure 2:**
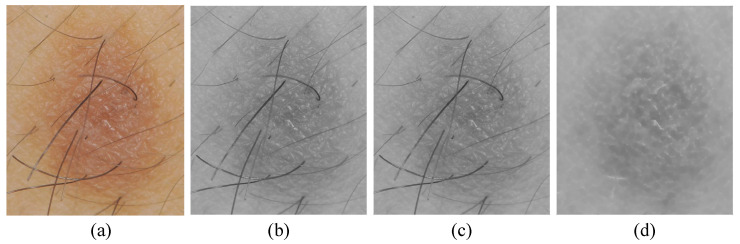
Main steps of preprocessing: (**a**) original image, (**b**) grayscale image, (**c**) noise removal with a median filter, and (**d**) hair artifacts removal with bottom-hat filtering.

For hair artifacts removal, the morphological bottom-hat filtering approach commonly used for increasing contrast in medical images [21] in the presence of shading is employed. This approach originally came from the usage of a cylindrical or parallelepiped structuring element function with a flat bottom. In a white background, a bottom-hat filter enhances black spots, subtracting the image’s morphological close from the image. The result is that neighboring pixels are properly connected and holes are filled up. High-frequency regions can effectively be inverted by the bottom-hat transform. Accordingly, the difference between processed image and original image will allow only the convex and concave features (instead of all features) of the image edge to be extracted. An example of hair artifact removal on dermoscopy images using morphological operations (e.g., median filter and bottom-hat filtering) can be seen in Figure 2.

#### Lesion Segmentation

Lesion image segmentation is a vital step in each CAD system for skin cancer, which mainly aims at the separation of skin lesion regions of interest (ROIs) from images. To this end, the process is initialized by the application of an automatic thresholding technique, such as the Otsu adaptive thresholding technique [22], to the R, G, and B image planes individually to extract the target lesion region from the pre-processed image. Then, binary masks for each plane are obtained and concatenated to create a final lesion mask. To improve segmentation accuracy, we utilize a 3-plane masking approach. The initially segmented image might also include several smaller blobs that are not skin lesions. A proper solution to this issue is applying morphological opening filtering [23] on the binarized image. Lastly, the final segmented area containing only the skin lesion can be determined by applying an iterative median filter technique to smooth the binary image using a series of gradually decreasing filter sizes (i.e., 7 × 7, 5 × 5, and 3 × 3).

**Figure 3:**
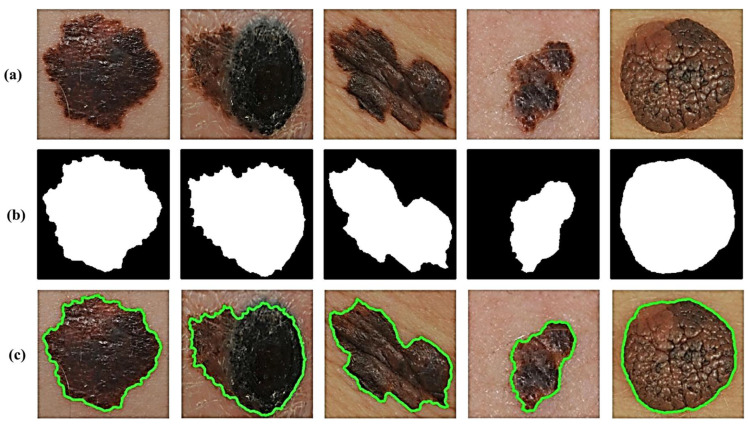
Results of lesion image segmentation: (**a**) Original image, (**b**) Binary Mask, and (**c**) Traced Lesion.

In addition, we take extra precautions such as adding two additional filters to avoid maintaining extremely small non-skin objects and, thus, ensure the reliable detection of accurate skin lesions of interest. This is generally achieved in two steps. The first step involves the removal of too-small objects from the binary image, while maintaining large objects in terms of size and shape by using an adaptive morphological open-close filter iteratively. In the second step, a so-called size filter is applied to remove objects whose size is lower than a given threshold. Experimentally, all spurious artifacts of a size less than 5% of the input image size are removed from the binary image. All image contours can then be found by using a modified canny edge detector [24] after erasing all irrelevant image components and isolated objects. The results in Figure 3 clearly show how the segmentation procedure allows for extremely accurate segmentation of skin lesions from the surrounding healthy skin regions.

### 3.2. Feature Extraction

Feature extraction process is very vital to extract the key features from the preprocessed skin lesions and uses the extracted features to achieve a most accurate representation of these lesions in order to visually comprehend the lesions’ morphological characteristics that necessarily help to differentiate melanoma from melanocytic nevi. In the proposed CAD methodology, we present a feature description framework involving several multiple feature descriptors (e.g., LBP, N-LBP, HOG, and CS-HOG) for building a robust CAD model for melanoma identification. The engineered feature descriptors are described in the following subsections.

#### 3.2.1. Local Binary Patterns (LBP)

The LBP operator that is an invariant texture measure derived from a general definition of texture in a local neighborhood has demonstrated excellent performance as a powerful tool for texture description due to its important properties, such as discriminative capability, computational simplicity, and its tolerance against monotonic illumination changes [25,26]. The LPB descriptors  [27] have been markedly exploited by the recent advances in facial recognition, biomedical image analysis, medical image retrieval, and motion analysis. In a specific square mask, the LBP algorithm thresholds the neighboring pixels based on the value of the center pixel, by performing a binary comparison between the central pixel and its neighbors. More formally, let us denote the gray value of the center pixel (x,y) in the image I(x,y) as $g_{c}$. In $g_{c}=I\left(x,y\right)$, let $g_{p}$ denote the gray values of the pixel *p* bin the neighborhood with radius *R* around the point (x,y),

(1) $$g_{p}=I\left(x_{p},y_{p}\right),\;p=0,1,\ldots ,P-1$$

where $x_{p}=-R\sin\left(2\pi /p\right)$ and $y_{p}=R\cos\left(2\pi /p\right)$. Assume the image I(x,y) has a texture *T* in a local neighborhood of a monochrome texture image that follows the joint distribution of gray values of P (P>1) pixels:

(2) $$T=t(g_{c},g_{0},g_{1},\ldots ,g_{p-1})$$

Then, without losing information, we extract the neighborhood’s center pixel:

(3) $$T=t(g_{c},g_{0}-g_{c},g_{1}-g_{c},\ldots ,g_{p-1}-g_{c})$$

Equation (3) can be factorized as follows, assuming the center pixel is statistically independent of the differences:

(4) $$T\approx t\left(g_{c}\right)t\left(g_{0}-g_{c},g_{1}-g_{c},\ldots ,g_{p-1}-g_{c}\right)$$

Most of the information in Equation (4) is in the joint distribution of differences by using vector quantization. The distribution can then be estimated as:

(5) $$T\approx t(s\left(g_{0}-g_{c}\right),s\left(g_{1}-g_{c}\right),\ldots ,s\left(g_{p-1}-g_{c}\right))$$

where

$$s\left(x\right)=\left\{\begin{array}{cc} 1 & ,\;x\geq 0\\ 0 & ,\;x<0 \end{array}\right.$$

This joint distribution provides a local binary pattern that is calculated by summing the threshold differences weighted by the power of two. By assigning a binomial factor $2^{p}$ for each sign $s(g_{p}-g_{0})$, a unique $LBP_{P,R}$ number that characterizes the spatial structure of the local image texture can be obtained as:

(6) $$LBP_{P,R}=\sum_{\begin{array}{c} p=0 \end{array}}^{p-1}s\left(g_{p}-g_{0}\right)2^{p}$$

Several textural features, such as the mean, standard deviation, energy, entropy, and image contrast, can be extracted by using Equation (6). In the presented work, the LBP values are calculated from grayscale values of skin lesions based on the relative grey values of the center pixel and the pixels in the neighborhood. From Equation (6), for 8 neighboring pixels, we have a histogram feature vector length of 256. In order to gain more details of crucial skin lesion regions from dermatoscopy images, we propose a modification in the original LBP algorithm (N-LBP) in term of the difference value between the center pixel and neighboring pixels, by increasing the number of neighboring pixels from 8 to 24. Hence, a more representative histogram descriptor built up of a feature vector of length $2^{24}=$ 16,777,216 would be obtained.

#### 3.2.2. Histogram of Oriented Gradients (HOG)

HOG descriptors that have been originally introduced by Dalal and Triggs in [28] for achieving a fast and accurate pedestrian detection in real-time, have recently received considerable attention from the computer vision and pattern recognition communities, due to their significant effectiveness and robustness characteristics in various domains, especially in real-world conditions. The data extracted with HOG have been shown to be a simple and effective way of describing the local appearance and shape characteristics of image objects using the distribution of local intensity gradients of direction edges.

The implementation process of the HOG extraction algorithm proceeds as follows. First, a given lesion image is divided into smallest regions (i.e., cells). Then, the horizontal Gx and vertical gradients Gy of each pixel in a cell are calculated:

(7) $$\begin{array}{ccc} Gx & = & I(x+1,y)-I(x-1,y)\\ Gy & = & I(x,y+1)-I(x,y-1) \end{array}$$

Thus, the gradient magnitude and orientation can be obtained by:

(8) $$\begin{array}{ccc} m(x,y) & = & \sqrt{{\left(Gx\right)}^{2}+{\left(Gy\right)}^{2}}\\ \theta (x,y) & = & \tan^{-1}\left(Gy/Gx\right) \end{array}$$

For each cell, a histogram with specified bins is created from the gradient orientations of the pixels within the cell. For histogram normalization, adjacent cells are combined into larger spatial regions, namely ‘blocks’, and the results are used to normalize all the cells in the block. The final HOG feature descriptor is formed as the concatenated 1D vector of the components of the normalized cell histograms from all of the block regions.

In our proposed methodology, an enhanced lesion image is typically divided into 8×8 cells and a gradient histogram is calculated for each cell. The calculation of histograms across a patch not only makes the descriptor representation more compact, but also makes it more noise resistant (i.e., less sensitive to noise). Due to individual gradient noise, we present a modification in the cell size parameter of HOG features (CS-HOG) to achieve higher detection rates. To gain an optimization procedure, an input lesion image is split into 2×2 cells, with a gradient histogram computed for each of the 2×2 cells. Furthermore, a 2×2 patch allows the histogram representation to be less prone to noise irrespective of individual gradient noise.

### 3.3. Feature Classification

In the subsection, the feature classification module of the presented automatic system for skin cancer detection is described. Broadly speaking, the main goal of the classification module is to classify skin lesions into two categories, benign skin tumors (nevus) or malignant tumors (melanoma). In this work, several supervised machine learning techniques (including SVM, kNN, and GAB) are explored to accurately predict the probability of skin cancer diagnosis. In the classification module, a machine learning (ML) model primarily depends on the availability of a set of previously labeled skin lesion images to be trained on. In this case, the set of labeled lesion images is termed ‘training set’ and the used learning strategy is termed ‘supervised learning’. The trained ML model is then used to predict the label of a new dataset of skin lesion images.

#### 3.3.1. Support Vector Machines (SVMs)

SVMs are supervised learning models widely used to perform classification and regression analysis, which have been initially developed for binary classification and later abruptly extended to a multi-class paradigm. In this work, SVM is employed for the classification of skin cancer into one of two diagnostic classes, melanoma or nevus. The basic idea of SVM is to map the training lesion data in the input space into a linear-separable higher-dimensional feature space using the kernel trick implicitly [29], and construct a separating hyperplane (or a set of hyperplanes) with a maximum margin. More formally, given training data $X={\left\{x_{i},y_{i}\right\}}_{i=1}^{N}$, with $x_{i}\in R^{d}$, where $x_{i}$ is an input feature vectors, and $y_{i}\in \left\{-1,+1\right\}$ is the class label of $x_{i}$, the main goal of SVM is to determine an optimal separating hyperplane (in terms of classification error and separation margin) that maximizes the margin between the two classes,

(9) $$f\left(x\right)=w^{T}\left(x\right)+b$$

where *w* and *b* are the weight vector and bias, respectively. SVMs use a kernel trick (in the current work, the non-linear Gaussian radial basis function (RBF) was employed in the numerical experiments) to map the original data points into the higher dimensional feature space. Thus, the optimal separating hyperplane is given as

(10) $$f\left(x\right)=w^{T}\varphi \left(x\right)+b$$

where $\varphi \left(x\right)$ is a non-linear kernel function. For a given test vector *x*, the decision function is defined by:

(11) $$f\left(x\right)=sign\left(w^{T}\varphi \left(x\right)+b\right)$$

#### 3.3.2. k-Nearest Neighbors (kNN)

In statistics, kNN is one of the best-known non-parametric and lazy learning models for classification; the term ‘non-parametric’ refers to the fact that there is no assumption for the underlying training data distribution. Furthermore, the kNN algorithm is simple, easy to implement, very efficient to train, and very fast for solving both classification and regression problems. Therefore, the kNN could truthfully be an optimal choice for any classification study, particularly when there is a little or no prior knowledge about the distribution of the data [30]. The kNN classification is one of the most popular distance-based algorithms, which focuses primarily on measuring the similarity or distance between the test samples and the training samples to decide the final classification output, using a specific similarity measure, e.g., Euclidean distance:

(12) $$d_{xy}=\sqrt{\sum_{i=1}^{n}{\left(x_{i}-y_{i}\right)}^{2}}$$

The investigations show that the performance of the kNN classifier heavily depends on the number of neighbors (*k*). The optimal choice of the *k* value is strongly data dependent. In general, a larger *k* value reduces the effects of noise, but makes the boundaries between the classes less distinct [31]. By using cross-validation, the best value of *k* can be selected, but there is unnecessary processing of the dataset for all possible values of *k*. There are a variety of techniques available for improving the performance and speed of a kNN classification. An approach is to select a subset of the training data, such that classification by the 1-NN rule approximates the Bayes classifier using the subset. This can yield significant speed improvements as *k* can now be limited to 1 and redundant data points are removed from the training set. These data modification techniques can greatly enhance the performance by eliminating points that result in misclassifications. The kNN algorithm follows the following main steps. First, in the training phase, both training examples and class labels of these examples are stored, where it is not allowed for missing and non-numeric data. In the classification stage, a test example is classified using the most frequent vote of its neighbors. Distances from the test sample to all stored training samples are calculated using a specific distance function or similarity measure. Additionally, the test example is selected, where *k* is prior defined as small integer. Finally, the majority class of this classifier is assigned to the test example. A pseudo code of the kNN algorithm is listed in Algorithm 1 below.
**Algorithm 1** Main steps in the kNN algorithm**Input:** Training samples *G*, test sample *g*, *K* **Output:** Class label of test sample 1: Measure the distance between *g* and each sample in *G* 2: Select *K* samples in *G* which are nearest to *g*; indicate the set by *P* (∈*G*) 3: Assign *g* the class that is the majority class

#### 3.3.3. Gentle AdaBoost (GAB)

Broadly speaking, boosting algorithms are an effective and simple method that widely applied in real-time systems for accurate prediction and improve classification error rate in machine learning. The main concept of boosting is to create strong learner “classifier” from weak learner “classifier” based on the weighted average values in addition to higher vote values. Different types of boosting algorithms are available, such as Adaptive boosting (AdaBoost), Gradient boosting, and XG Boost algorithms [32]. The AdaBoost algorithm was introduced by Freund and Schapire [33] in 1995 and refers to a specific way of training a boosted classifier. The Real AdaBoost (RAB), LogitBoost (LB), and Gentle AdaBoost (GAB) are variants of the AdaBoost algorithm.

Due to the fact that the GAB algorithm is more robust in the presence of noisy training data and highly resistant to overfitting (outliers), the GAB algorithm has better performance than other boosting methods for various types of real-world problems of machine learning, e.g., pattern classification, face recognition, and text categorization. The authors in [34] endeavoured to find a stationary point instead of pursuing rigorous optimization through the gradual optimization of each applied Newton step. The application of weighted least squares regression resulted in a high performance of GAB results for exponential error function reduction compared with other options [35]. Furthermore, while the normalizing function of Real AdaBoost is given [34], Real AdaBoost and Gentle AdaBoost do not normalize all learners weighted in the same way. The main steps of the GAB algorithm are presented in Algorithm 2 below. In GAB, the following function is used to update the weighted probability class:

(13) $$F_{m}\left(x\right)=P_{w}\left(y=1|x\right)-P_{w}\left(y=-1|x\right)$$

**Algorithm 2** The GentleAdaBoost algorithm**Input:** Training data $x_{i}$; *i*∈[1, *N*] with corresponding class labels $y_{i}$; *i*∈[1, *N*] **Output:** A robust classifier F(x) 1: Initialize weights $w_{i}$ = 1/*N*, and F(x) = 0 2: For *m* = 1, 2, 3,…, M do 3: Train $f_{m}\left(x\right)$ by weighted least square of $y_{i}$ to $x_{i}$ with weights $w_{i}$ 4: Update F(x) = F(x) + $f_{m}\left(x\right)$ 5: Update the weights by $w_{i}=w_{i}\exp(-y_{i}f_{m}\left(x_{i}\right))$ and normalize the weights $\sum_{i=1}^{N}$$w_{i}$ = 1 6: end for 7: Final Classifier F(x)= sign [$\sum_{m=1}^{M}$$f_{m}\left(x\right)$]

## 4. Experimental Results and Analysis

In this section, we demonstrate the effectiveness of the proposed CAD framework for skin lesions with several extensive experiments conducted on the publicly available MED-NODE skin lesion dataset. The dataset contains a total of 170 dermatoscopic skin lesion images (70 melanomas and 100 nevi) obtained from the digital dermatoscopic image archive of the Department of Dermatology, University Medical Center Groningen (University of Groningen, The Netherlands). The skin lesion images were captured with a Nikon D3 or Nikon D1x body and a Nikon 2.8G/105 mm f micro-Nikkor lens. The distance between the lens and the lesion is around 33 cm in 95% of instances. Moreover, two Multiblitz Variolite 600 flash units with a color temperature of 5200 Kelvin were used to create the lighting settings. All the images are part of a much bigger dermatology digital library that contains over 50,000 images of various lesions. A dataset with entirely unidentifiable patient cases is picked at random to create the MED-NODE expert system. The skin lesion images in the dataset are grouped in single binary class (i.e., only superficial spreading melanoma and nevi, excluding Acral lentiginous/Nodular melanomas, Seborrhoeic Warts, and Spitz nevi). Only pigmented skin lesion images were created from Caucasian patients origin who represent the majority of the Netherlands’ population.

In order to improve the overall classification results and avoiding classification bias (overoptimistic impact) for imbalanced data, a random over-sampling technique [36] is applied, where the existing melanoma samples are randomly replicated (30 samples) until the class distribution is balanced. For the sake of computational manageability, all skin lesion images in the dataset are first converted into grayscale and then resized to 150×150 pixels to have a uniform size. In order to guarantee the validity of the results, the leave-one-out cross-validation technique is applied, where the original dataset is first sorted and then randomly split into k-folds (k is set experimentally to 10). Thus, the performance of the classification models is evaluated on each of the 10 folds separately, after being trained on the remaining 9 folds.

For performance analysis of the classification models, the confusion matrix (i.e., a cross-plot) is applied to summarize classifier performance, which reflects the relation between actual and predicted values and it is mainly made up of a 2×2 table of four components (representation of the predicted result and the actual value), namely True Positive (TP), True Negative (TN), False Positive (FP), and False Negative (FN) defined as follows:

– True Positive (TP) denotes the positive cases that are correctly predicted as positive.
– True Negative (TN) denotes the negative cases that are correctly predicted as negative.
– False Positive (FP) denotes the positive cases that are incorrectly predicted as negative.
– False Negative (FN) denotes the negative cases that are incorrectly predicted as positive.

**Figure 4:**
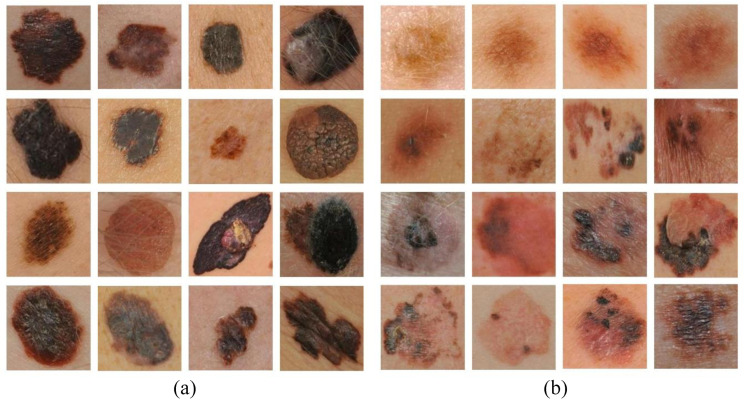
Examples of (**a**) correctly classified and (**b**) misclassified dermoscopy images.

In Figure 4, examples of correctly classified and misclassified dermoscopy image samples are shown.

**Figure 5:**
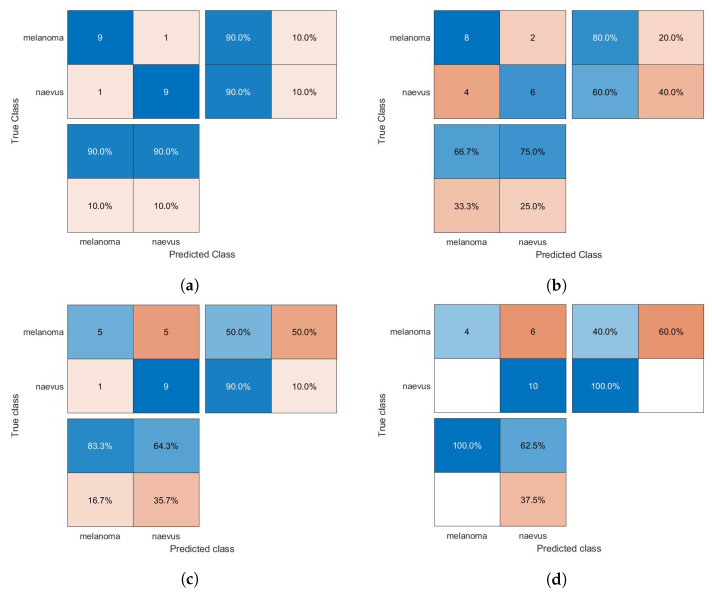
Confusion matrices for the SVM classifier trained using (**a**) HOG, (**b**) CS-HOG, (**c**) LBP, and (**d**) N-LBP features.

**Figure 6:**
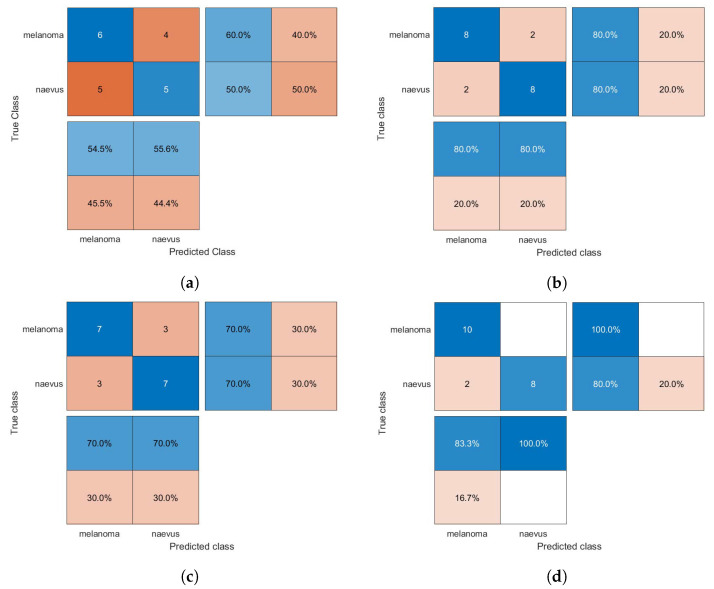
Confusion matrices for the GAB classifier trained using (**a**) HOG, (**b**) CS-HOG, (**c**) LBP, and (**d**) N-LBP features.

**Figure 7:**
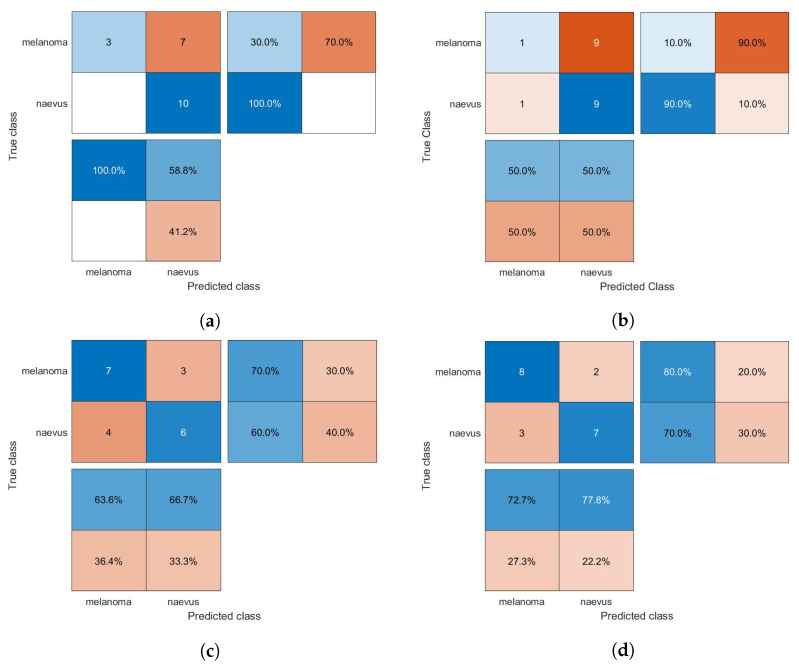
Confusion matrices for the kNN classifier trained using (**a**) HOG, (**b**) CS-HOG, (**c**) LBP, and (**d**) N-LBP features.

The confusion matrices of the classification results for the SVM, GAB, and kNN models are shown in Figure 5, Figure 6 and Figure 7, respectively, using different feature selection methods (i.e., HOG, CS-HOG, LBP, and N-LBP).

The proportions mentioned above of true and false results can be successfully utilized to calculate several diagnostic performance statistics, such as accuracy (AC), sensitivity (SE), specificity (SP), positive predictive value (PPV), and negative predictive value (NPV), as follows:

(14) $$\begin{array}{ccc} AC & = & \frac{TP+TN}{TP+TN+FP+FN}\times 100\%\\ SE & = & \frac{TP}{TP+FN}\times 100\%\\ SP & = & \frac{TN}{TN+FP}\times 100\%\\ PPV & = & \frac{TP}{TP+FP}\times 100\%\\ NPV & = & \frac{TN}{TN+FN}\times 100\% \end{array}$$

Table 1 reports the performance of the SVM, GAB, and kNN classification models in terms of AC, SE, SP, PPV, and NPV, based on different feature descriptors, namely HOG, CS-HOG, LBP, and N-LBP.

From the results presented in Table 1, one can observe that the GAB model yielded the highest AC value of 94%, when using N-LBP features. For sensitivity, an optimum SE value of 100% was reached by both the SVM and GAB models, when using N-LBP and CS-HOG features, respectively. Similarly, both the SVM and kNN showed extraordinary performance with optimum SP and PPV of 100%, when using N-LBP and HOG/CS-HOG features, respectively. Additionally, both the GAB and SVM models have the best performance in term of NPV (100%), with N-LBP and CS-HOG features, respectively.

To validate the performance of the proposed diagnostic framework, the obtained results are extensively compared with those of similar existing state-of-the-art techniques. A summary of the performance comparison to other techniques is provided in Table 2.

The qualitative comparison in the above table demonstrates that the presented automated method for melanoma diagnosis performs favorably against various competing methods in most of performance metrics. All the algorithms and classification routines employed in this work have been implemented in MATLAB software (version R2019a, MathWorks) on a PC with an Intel(R) Core(TM) i7 CPU-2.60 GHz processor, 8 GB RAM, running a Windows 10 Professional 64-bit operating system.

## 5. Conclusions

In this work, we have introduced an innovative framework for an automated CAD system for melanoma skin cancer, by utilizing two representative local descriptors (HOG and LBP) and their extensions and applying three machine learning classification models (SVM, kNN, and GAB). An extensive set of experimental evaluations performed by using 10-fold cross-validation on the publicly available MED-NODE dermoscopy image dataset has demonstrated that the presented CAD framework can exhibit consistently superior performance over several existing state-of-the-art baselines on melanoma recognition in terms of accuracy, sensitivity, and specificity. Regarding future work, our goal is two-fold. On the one hand, we intend to improve pre-processing stage to extract a most informative set of local features from skin lesions and on the other hand, we plan to develop a CAD architecture which fuses an innovative preprocessing strategy with a modern deep learning methodology for skin lesion classification in order to achieve better findings.

## Figures and Tables

**Table 1:** Performance analysis of classifiers based on CS-HOG and HOG features.

Models	AC (%)	SE (%)	SP (%)	PPV (%)	NPV (%)
HOG + SVM	80	80	80	80	80
CS-HOG + SVM	85	100	70	76.9	100
LBP + SVM	70	50	90	83.3	64.3
N-LBP + SVM	70	40	100	100	62.5
HOG + GAB	90	90	90	90	90
CS-HOG + GAB	80	80	80	80	80
LBP + GAB	70	70	70	70	70
N-LBP + GAB	94	100	92	83.3	80
HOG + kNN	65	30	100	100	58.8
CS-HOG + kNN	55	10	100	100	52.6
LBP + kNN	65	70	60	63.6	66.7
N-LBP + kNN	75	80	70	72.7	77.8

**Table 2:** Qualitative performance comparison of the proposed framework with various state-of-the-art methods using the public MED-NODE skin lesion dataset.

Method	AC (%)	SE (%)	SP (%)
Our method	94.0	100	92.0
Jeyakumar et al. [37]	97.1	96.8	96.0
Singh et al. [4]	81.8	70.0	82.6
Mukherjee et al. [38]	87.2	87.4	86.8
Mukherjee et al. [39]	85.9	86.2	85.5
Giotis et al. [9]	81.0	80.0	81.0

## Data Availability

Not applicable.
